# Emergent electric field control of phase transformation in oxide superlattices

**DOI:** 10.1038/s41467-020-14631-3

**Published:** 2020-02-14

**Authors:** Di Yi, Yujia Wang, Olaf M. J. van ʼt Erve, Liubin Xu, Hongtao Yuan, Michael J. Veit, Purnima P. Balakrishnan, Yongseong Choi, Alpha T. N’Diaye, Padraic Shafer, Elke Arenholz, Alexander Grutter, Haixuan Xu, Pu Yu, Berend T. Jonker, Yuri Suzuki

**Affiliations:** 1 Geballe Laboratory for Advanced Materials, Stanford University, Stanford, CA 94305 USA; 2 State Key Laboratory of Low Dimensional Quantum Physics and Department of Physics, Tsinghua University, Beijing, 100084 China; 3 Materials Science and Technology Division, US Naval Research Laboratory, Washington, DC 20375 USA; 4 Department of Materials Science and Engineering, University of Tennessee, Knoxville, TN 37996 USA; 5 National Laboratory of Solid-State Microstructures, College of Engineering and Applied Sciences, and Collaborative Innovation Center of Advanced Microstructures, Nanjing University, 210093 Nanjing, China; 6 Department of Applied Physics, Stanford University, Stanford, CA 94305 USA; 7 Department of Physics, Stanford University, Stanford, CA 94305 USA; 8 Advanced Photon Source, Argonne National Laboratory, Argonne, IL 60439 USA; 9 Advanced Light Source, Lawrence Berkeley National Laboratory, Berkeley, CA 94720 USA; 10 Cornell High Energy Synchrotron Source, Cornell University, Ithaca, NY 14853 USA; 11 NIST Center for Neutron Research, National Institute of Standards and Technology, Gaithersburg, MD 20899-6102 USA; 12 Frontier Science Center for Quantum Information, Beijing, 100084 China; 13 RIKEN Center for Emergent Matter Science (CEMS), Saitama, 351-0198 Japan

**Keywords:** Ferroelectrics and multiferroics, Ferromagnetism, Electronic properties and materials, Surfaces, interfaces and thin films

## Abstract

Electric fields can transform materials with respect to their structure and properties, enabling various applications ranging from batteries to spintronics. Recently electrolytic gating, which can generate large electric fields and voltage-driven ion transfer, has been identified as a powerful means to achieve electric-field-controlled phase transformations. The class of transition metal oxides provide many potential candidates that present a strong response under electrolytic gating. However, very few show a reversible structural transformation at room-temperature. Here, we report the realization of a digitally synthesized transition metal oxide that shows a reversible, electric-field-controlled transformation between distinct crystalline phases at room-temperature. In superlattices comprised of alternating one-unit-cell of SrIrO_3_ and La_0.2_Sr_0.8_MnO_3_, we find a reversible phase transformation with a 7% lattice change and dramatic modulation in chemical, electronic, magnetic and optical properties, mediated by the reversible transfer of oxygen and hydrogen ions. Strikingly, this phase transformation is absent in the constituent oxides, solid solutions and larger period superlattices. Our findings open up this class of materials for voltage-controlled functionality.

## Abstract (web summary)

Many complex oxides already have rich functional behavior but oxide heterostructures can exhibit new emergent properties. Yi et al. show that LSMO/SIO superlattices have a reversible electric-field-controlled structural phase transition that is not present in the constituent materials.

## Introduction

Electrolytes have been widely exploited to control electronic, magnetic, and optical properties of materials when large electric fields are desired. Under electrolytic gating, changes in these properties have largely been attributed to changes in carrier concentration^1–5^. Recent advances have shown that electrolytic gating can also lead to the transfer of oxygen and hydrogen ions that modifies the structure of transition metal oxides (TMOs), leading to more dramatic modulation of their physical properties^6,7^. To fully exploit this mechanism, materials that can be electrically switched from one crystalline phase to another, with distinct physical properties, are highly desirable. Although ion transfer has been reported in many single-phase TMOs^8–18^, reversible, electric-field-controlled transformation between distinct crystalline phases at room-temperature (RT) has been limited to a few systems, including binary oxides VO_2_^8^ and WO_3_^12,13^, and perovskite oxides SrCoO_3-*δ*_^14^ and SrFeO_3-*δ*_^17^ that exhibit topotactic transformations. Among these systems, coupled electronic, magnetic, and optical phase transitions have only been demonstrated in SrCoO_3-*δ*_ thus far^14^. The scarcity of single-phase oxides as candidate materials demands strategies to develop tunable materials to this end.

Here we show that digital oxide superlattices can be modulated by electrolytic gating to trigger reversible coupled phase transformations at RT. Using in situ X-ray diffraction (XRD), we find that electrolytic gating induces a reversible structural transformation in superlattices comprised of alternating one-unit-cell of La_0.2_Sr_0.8_MnO_3_ and SrIrO_3_. By contrast, the constituent oxides, their solid solutions and larger period superlattices do not show such behavior. Through secondary-ion mass spectrometry (SIMS), we confirm the transfer of both hydrogen and oxygen ions in the [(La_0.2_Sr_0.8_MnO_3_)_1_(SrIrO_3_)_1_]_20_ superlattices. This transformation leads to a reversible metal-insulator transition accompanied by optical and magnetic transitions. More specifically, ferromagnetism with perpendicular magnetic anisotropy can be suppressed entirely in conjunction with metal-insulator and optical transitions. Together these results demonstrate the discovery of a class of oxides that exhibits reversible and coupled phase transformations with rich functionalities through electrolyte-based ionic control.

## Results

### Synthesis of digital superlattices and reference films

**Fig. 1:**
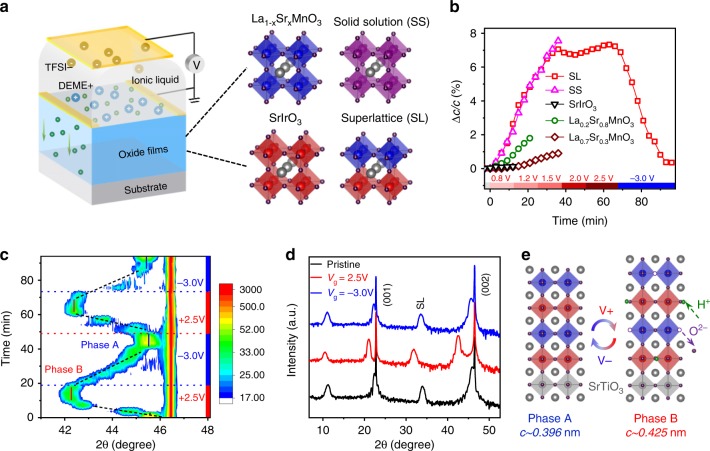
Electric-field control of structural transition. **a** Schematic of ionic liquid gating (ILG) that induces the ion transfer between oxides and ionic liquid. Four types of oxides were studied, i.e. manganate (La_1-x_Sr_x_MnO_3_) and iridate (SrIrO_3_) films, solid solution (Sr(Mn_0.5_Ir_0.5_)O_3_) films and superlattices ([(La_0.2_Sr_0.8_MnO_3_)_*m*_(SrIrO_3_)_*m*_]_*n*_) on SrTiO_3_ substrates. **b** Modulation of out-of-plane lattice constant (∆*c*/*c*) during voltage cycling (with incremental positive voltages to +2.5 V and −3.0 V) for different samples (SS refers to Sr(Mn_0.5_Ir_0.5_)O_3_ and SL refers to [(La_0.2_Sr_0.8_MnO_3_)_1_(SrIrO_3_)_1_]_20_). The lattice constant was extracted from in situ measurements of the (002) peak position. The end of the curves for all samples except the superlattice corresponds to phase decomposition above certain voltages. **c** In situ X-ray diffraction results of the superlattices around the (002) peak during ILG in repeated voltage cycling (sequence of +2.5 V, −3.0 V, +2.5 V, −3.0 V), showing a reversible electric-field-controlled transformation between two phases of the superlattices. **d** Full-range X-ray diffraction of a superlattice in as-grown pristine state, positively gated state and reversibly gated state. **e** Schematic of the reversible phase transformation mediated by dual-ion (both H^+^ and O^2−^) transfer.

Perovskite TMOs of the ABO_3_ form provide excellent candidates for strong electrochemical response due to the wide range of possible A and B cations in conjunction with the role of transition metal cation and oxygen anion (B–O) bonds for tunable electronic structure^19^. In addition, the correlated *d* electrons of B cations give rise to strongly coupled physical properties. Here we studied epitaxial heterostructures of the 3*d* TMOs La_1-x_Sr_x_MnO_3_ that show rich physics and the 5*d* TMO SrIrO_3_, which has attracted recent research interests to explore exotic properties arising from spin-orbit coupling^20–26^. To elucidate the role of B-site cation ordering, we synthesized superlattices comprised of La_0.2_Sr_0.8_MnO_3_ and SrIrO_3_ ([(La_0.2_Sr_0.8_MnO_3_)_*m*_(SrIrO_3_)_*m*_]_*n*_ with *m* = 1, 2, 4), in situ monitored by using reflection high-energy electron diffraction (RHEED) (Supplementary Fig. 1). In addition, we also synthesized single-phase films of La_0.2_Sr_0.8_MnO_3_, La_0.7_Sr_0.3_MnO_3_, and SrIrO_3_ and B-site disordered solid solution films of Sr(Mn_0.5_Ir_0.5_)O_3_ to shed light on the design strategies. Figure 1a shows the four types of materials. All samples are 40 unit-cells (~16 nm) thick and were grown on (001)-oriented SrTiO_3_ (STO) substrates. XRD shows that all samples exhibit good epitaxy and excellent crystallinity (Supplementary Fig. 2). Our previous studies have shown the excellent unit-cell layering of the superlattices by using transmission electron microscopy^22,27^. All samples were electrolytically gated with the ionic liquid DEME^+^–TFSI^−^ on the film surface, and voltages were applied across the electrolyte using electrical contacts to the top electrode and film surface (Fig. 1a).

### Reversible structure transformation

To directly observe structural changes induced by ionic liquid gating (ILG), we first performed in situ XRD measurements during ILG at RT. First of all, upon ILG, in situ XRD measurements show large changes in the out-of-plane lattice parameter (*∆c/c*) as high as 7% in [(La_0.2_Sr_0.8_MnO_3_)_1_(SrIrO_3_)_1_]_20_ superlattices while single-phase films show much smaller changes and undergo irreversible structural changes at relatively small bias voltages. Secondly, only [(La_0.2_Sr_0.8_MnO_3_)_1_(SrIrO_3_)_1_]_20_ superlattices show reversible structural transformations with the application of a reverse voltage. Figure 1b shows the time dependence of *∆c/c* for different types of samples during voltage cycling; the (002) peak was monitored in situ while incremental positive voltages were applied up to 2.5 V and then reversed to negative −3 V (Supplementary Fig. 2b–f). For manganate films, the maximum *∆c/c* ratio is achieved at around 2% (1%) for La_0.2_Sr_0.8_MnO_3_ (La_0.7_Sr_0.3_MnO_3_) before the (002) peak disappears, and the process is irreversible even with the application of −3 V. For SrIrO_3_ films, the (002) peak quickly decays at a lower voltage (~1.2 V). This may be attributed to the fact that the perovskite phase of SrIrO_3_ is thermodynamically metastable in bulk and only epitaxially stabilized on STO substrates. Intriguingly, the maximum *∆c/c* significantly increases to about 7% in solid solution films that have a random mixture of Mn and Ir cations. However, we find that the (002) peak in solid solution films also disappears below 2 V and that this change is irreversible.

By contrast, the B-site ordered [(La_0.2_Sr_0.8_MnO_3_)_1_(SrIrO_3_)_1_]_20_ superlattices show a large reversible structural transformation. As shown in Fig. 1b, the lattice expansion *∆c/c* of superlattices is similar to that of solid solution films (~7%) up to bias voltages of 2 V. Beyond 2 V, the (002) peak of the superlattices retains the 7% expansion which can be reversed with negative voltages. Notably, as the period of superlattices (*m*) increases, the lattice expansion decreases and the change becomes irreversible (Supplementary Fig. 3), further highlighting the importance of B-site cation ordering at the atomic scale. For the rest of this paper, the term superlattice is used to refer to [(La_0.2_Sr_0.8_MnO_3_)_1_(SrIrO_3_)_1_]_20_ superlattices unless otherwise stated.

Figure 1c shows the in situ XRD results of the superlattices during repeated cycling. With +2.5 V, the (002) peak (around 45.7°, corresponding to *c* ~ 0.396 nm) gradually shifts towards lower angles, indicating the elongation of the *c* lattice parameter. Eventually, a new diffraction peak develops at around 42.4° (corresponding to *c* ~ 0.425 nm), revealing the emergence of a new phase (denoted as phase B). By reversing the voltage to −3 V, the superlattice gradually returns to the original phase (denoted as phase A). Notably, the ILG-induced changes are nonvolatile, as phase B is stable over an extended time at ambient conditions (Supplementary Fig. 4a). Thus, we can characterize the two phases by ex situ probes. Figure 1d shows full-range XRD of the superlattices, showing good epitaxy and cation ordering (deduced from pronounced satellite peaks) in both phases. Reciprocal space mapping shows that both phases are coherently strained by the underlying substrate (Supplementary Fig. 5).

### Voltage-driven dual-ion transfer

**Fig. 2:**
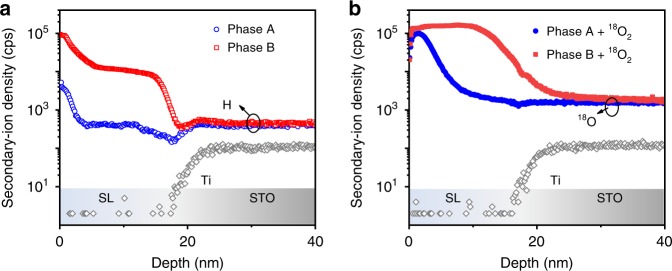
Chemical characterization. **a** Depth profiles of H and Ti signals in the two phases of the superlattices, measured by using secondary-ion mass spectrometry. The H signal was obtained in phase A and B of the superlattices stabilized by ILG. The Ti signal from the substrate indicates the position of the interface between substrate and superlattice. **b** Depth profiles of ^18^O and Ti signals in the two phases of the superlattices after thermal annealing in ^18^O_2_. To measure ^18^O signal, the superlattices were first stabilized in phase A and B under ILG, and then thermally annealed in ^18^O_2_ gas.

To identify whether the transfer of ions plays a role, we employed SIMS to measure ex situ the depth profiles of oxygen and hydrogen. It is known that ILG can induce changes in both oxygen and hydrogen stoichiometries in oxides, and one possible origin is the electrolysis of residual water^10,14^. To probe hydrogen ions, we stabilized two superlattices with phases A and B by ILG. As shown in Fig. 2a, SIMS results of the superlattice in phase B (red) reveal a significantly higher concentration of hydrogen than that of phase A (blue), confirming the incorporation of hydrogen ions in phase B. To explore the possibility of oxygen ion extraction (oxygen vacancy creation), we first stabilized two superlattices with phases A and B by ILG. Then both samples were thermally annealed in ^18^O_2_ gas (Supplementary Fig. 4b). We find that the phase B sample after thermal annealing in ^18^O_2_ shows a dramatic increase in ^18^O signal throughout the entire sample, while the phase A sample only shows an increase near the surface (Fig. 2b). Although the depth profile of the ^18^O signal does not directly measure the amount of oxygen vacancies, the additional incorporation of ^18^O throughout the entire phase B sample indicates that ILG creates oxygen vacancies in phase B (Supplementary Fig. 4c). It is noted that the SIMS results are consistent with polarized neutron reflectivity results, revealing the accumulation of oxygen vacancies and hydrogen ions in phase B (Supplementary Fig. 6). Therefore, we demonstrate that the phase transformation is mediated by voltage-driven dual-ion transfer, characterized by the exchange of both oxygen and hydrogen ions^14^.

### Valence modulation

**Fig. 3:**
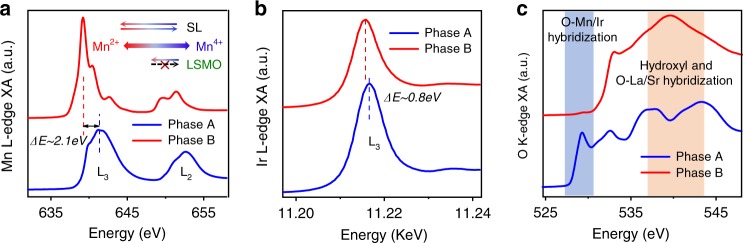
Valence characterization. X-ray absorption (XA) spectra of **a** Mn L-edge, **b** Ir L_3_-edge, and **c** oxygen K-edge of the two phases. Figure 3a inset shows the valence changes of the superlattices and La_0.2_Sr_0.8_MnO_3_ films under ILG. The shifts of absorption peaks at the Mn L-edge (**a**) and Ir L_3_-edge (**b**) reveal a significant decrease of valence for both Mn and Ir cations in phase B. The oxygen K-edge results (**c**) show a full suppression of hybridization between oxygen 2*p* orbitals and Mn/Ir *d* orbitals, and the appearance of features associated with hydroxyl bonds in phase B.

The large structural changes induced by ILG should be accompanied by changes in cation valence and oxygen bonding. To probe valence changes, we carried out ex situ X-ray absorption spectroscopy (XAS) at the Mn L-edge, Ir L-edge, and O K-edge. The absorption peak at the Mn-L edge (Fig. 3a and Supplementary Fig. 7a–c) shifts to lower energy by ~2.1 eV when comparing phase B to phase A, indicating a substantial decrease in Mn valence^28^. By comparing peak positions and multiplet features to references (Supplementary Fig. 8), we find that the Mn cations are close to +3.5 oxidation state in phase A and are dominated by +2 oxidation state in phase B. It is noted that the change of Mn valence in the superlattices is much larger than that in La_0.2_Sr_0.8_MnO_3_ films under ILG (Supplementary Fig. 9), consistent with the magnitude of the structural change. Complementary results at the Ir-L_3_ edge (Fig. 3b and Supplementary Fig. 7d) also show a clear shift towards lower energy in phase B, revealing a large decrease of the Ir oxidation state^29^. Moreover, the oxygen K-edge results (Fig. 3c) exhibit a complete suppression of the spectral feature associated with the hybridization of oxygen and Ir/Mn cations in phase B, confirming impact on the valence state. We also note that distinct XAS features around 540 eV were observed between two phases of the superlattices, which are known to indicate the presence of hydroxyl groups and are consistent with the SIMS results^30^.

### Voltage control of physical properties

**Fig. 4:**
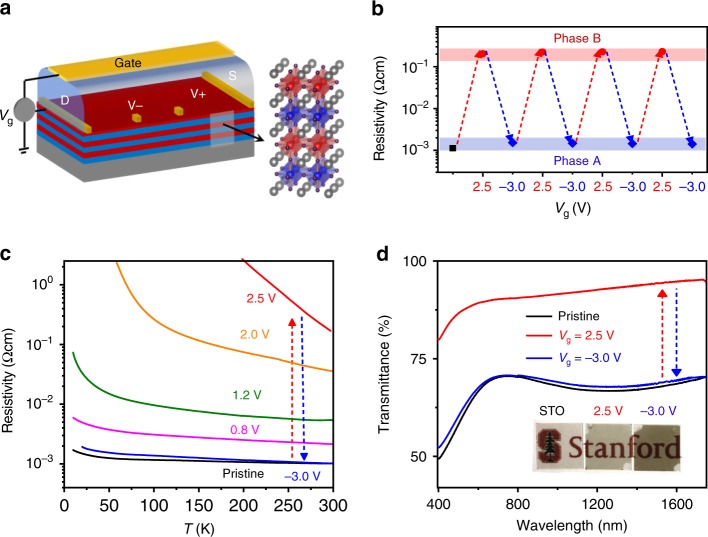
Electric-field control of resistivity and electrochromic effect. **a** Schematic of the device for in situ measurement of the transport properties under ILG. **b** Reversible modulation of resistivity between the two phases during repeated voltage cycling at room temperature. **c** Temperature dependence of resistivity of the superlattice during voltage cycling (incremental positive voltages to +2.5 V and −3 V). **d** Optical transmittance spectra of a superlattice during voltage cycling. Inset shows the photographs of a double-side polished SrTiO_3_ substrate and superlattices that are stabilized in the two phases by ILG.

Strikingly the large structural and chemical changes are accompanied by a concurrent metal-insulator transition, suppression of ferromagnetism and enhancement of optical transparency. We first studied the transport properties under ILG (Fig. 4a). The resistivity changes between the two phases are reversible during repeated cycling at RT (Fig. 4b). As shown in Fig. 4c, the superlattice in phase A shows the lowest resistivity and a weak temperature dependence. With increasing positive voltage, the resistivity gradually increases and eventually saturates in a highly insulating state of phase B (Supplementary Fig. 10). The resistivity changes by about two orders of magnitude at RT and three orders of magnitude around 200 K. The large tuning range and high reversibility of resistivity, mediated by voltage-driven ion transfer, make the superlattices of potential interest in resistive random-access memory devices^31^, electrochemical sensors^15^, and neuromorphic computing^32^.

The metal-insulator transition is accompanied by a significant modulation of optical transparency at visible wavelengths under ILG, showing an electrochromic effect in the superlattices. Figure 4d shows ex situ optical transmittance measurements in the visible and near-infrared regions. These measurements exhibit a reversible modulation of optical transmittance around 25–30% over the entire wavelength (400–1800 nm) between the two phases. This modulation can also be directly observed by eye, noting the strong opacity changes between the two phases (Fig. 4d inset). This electrochromic effect may find applications in smart windows^33^.

**Fig. 5:**
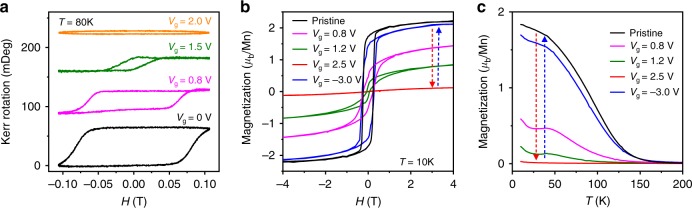
Electric-field control of magnetic properties. **a** Magnetic hysteresis loops of a superlattice under ILG, probed with in situ magneto-optic Kerr effect (MOKE). An offset is applied for illustration. **b** Magnetic hysteresis loops and **c** temperature dependence of magnetization of the superlattice during voltage cycling, probed with ex situ SQUID magnetometry. All measurements were performed along the out-of-plane direction, which is the magnetic easy axis due to interface perpendicular magnetic anisotropy. A field of 0.2 T was applied to obtain results in (**c**).

The most intriguing effect due to ILG was found in the suppression of ferromagnetism and perpendicular magnetic anisotropy (PMA). We note that the PMA, which is challenging to realize, is an emergent interfacial phenomenon in the superlattices^27^. The change of magnetism was confirmed by both in situ and ex situ magnetic characterization. The in situ measurements exploited the magneto-optic Kerr effect (Fig. 5a and Supplementary Fig. 11) and showed that ILG can fully suppress ferromagnetism with strong PMA. To quantify the changes over a wider temperature range, we carried out ex situ measurements by using SQUID magnetometry (Fig. 5b, c). When the superlattice is in phase A, a ferromagnetic ground state is stabilized with saturation magnetization M_s_ ~ 2 *µ*_*B*_/Mn, magnetic easy axis along the out-of-plane direction and Curie temperature (T_c_) around 150 K. With increasing positive voltage, both M_s_ and T_c_ decrease until the ferromagnetic transition is fully suppressed when the superlattice is in phase B. The suppression of ferromagnetism is also highly reversible after repeated cycling (Supplementary Fig. 12). Voltage-controlled PMA has been shown to be critical in developing magnetic memory devices with high density, good stability, and low power-consumption^34,35^.

## Discussion

By combining multiple in situ and ex situ probes, we have shown that the digital superlattices with atomic layering structure provide good candidates for electrolyte-based ionic control with both wide tunability and good reversibility. The structural and chemical characterization clearly show a reversible, electric-field-controlled transformation between two crystalline phases of the superlattices at RT, with distinct lattice parameters, chemical stoichiometries and valences, as schematically shown in Fig. 1e. More specifically, phase A of the superlattices is close to the as-grown state in terms of the lattice parameter (*c* ~ 0.396 nm) and chemical stoichiometry (close to ABO_3_). On the other hand, phase B shows a largely expanded lattice (*c* ~ 0.425 nm) and a massive loss (gain) of oxygen (hydrogen) ions (H_*x*_ABO_3-*δ*_). Although it is challenging to precisely quantify the local concentration of ions, we note that the magnitude of lattice expansion (~7%) and valence change are comparable to the highest reported values in singe-phase perovskite TMOs (e.g., H_*x*_SrCoO_3-*δ*_ with large transfer of hydrogen (*x* ~ 1) and oxygen (*δ* ~ 0.5) ions)^14^. This phase transformation is accompanied by simultaneous modulation of the electronic, optical, and magnetic properties.

Our results have revealed insights into the strategies to develop complex oxides for electrolyte-based ionic control. Firstly, as compared to single-phase constituent oxides, both superlattices and solid solution films exhibit giant structural changes with the application of positive voltages. This result indicates that the intermixing of 3*d* and 5*d* transition metal cations can modify the enthalpy of formation energy and diffusivity of ions (oxygen vacancies and hydrogen ions)^36^. More importantly, in order to achieve wide tunability and high reversibility, candidate materials need to be transformed into other crystalline phases after massive ion transfer, rather than undergoing phase decomposition by electrolytic gating. Our results have shown that a new crystalline phase only appears in the [(La_0.2_Sr_0.8_MnO_3_)_1_(SrIrO_3_)_1_]_20_ superlattices after ion transfer and not in the parent compounds, solid solutions or larger period superlattices, thereby highlighting the critical role of B-site cation ordering at the atomic scale.

Similarities can be drawn between these atomically layered (La_0.2_Sr_0.8_MnO_3_)_1_(SrIrO_3_)_1_ superlattices and single-phase oxides SrCoO_3-*δ*_^14^ and SrFeO_3-*δ*_,^17^ where structural phase transformations are brought about by electrolytic gating. Both SrCoO_3-*δ*_ and SrFeO_3-*δ*_ show a topotactic transformation into a vacancy-ordered brownmillerite phase after the transfer of ions, thus preserving the lattice framework without losing the crystallographic orientation and lattice structure^14,17,37^. Although single-phase manganate and iridate do not show this transformation at room-temperature as shown in Fig. 1b and Supplementary Fig. 2, the digital superlattices can provide a lattice framework to facilitate a structural phase transition. This is closely related to the unit-cell layered structure of the superlattices, which leads to the different formation energies of ions depending on the local chemical environment.

**Fig. 6:**
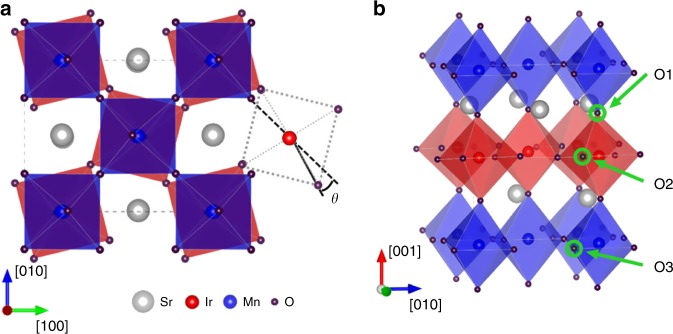
DFT calculations. **a** Top view of the superlattice without ionic defects. The IrO_6_ and MnO_6_ octahedra of the superlattice show different magnitudes of rotation. The calculated rotation angle (*θ*) along the c axis ([001]) is about 14.5° and 2.9° for IrO_6_ and MnO_6_ octahedra, respectively. **b** Side view of the superlattice with the three kinds of oxygen vacancy sites. Due to the atomically layered structure, three distinct oxygen sites were considered, labelled as O1 (in the SrO layer), O2 (in the IrO_2_ layer) and O3 (in the MnO_2_ layer). Further results on hydrogen interstitial are included in the Supplementary Note 13.

To reveal the correlation between the formation energy of ions and local chemical environment, we performed first-principles calculations. For simplicity, we use the supercell that is composed of alternating one-unit-cell of SrIrO_3_ and SrMnO_3_ in the calculations (details are included in the Methods section and Supplementary Note 13). Figure 6a shows the calculated crystal structure of the superlattice without ionic defects, showing different rotation angles of IrO_6_ and MnO_6_ octahedra along the **c** axis. Subsequently, an oxygen vacancy or a hydrogen interstitial is introduced into the supercell and different possible positions have been considered. For instance, given the unit-cell layered structure, three types of oxygen vacancy positions were tested, i.e., in the SrO layer, in the IrO_2_ layer, or in the MnO_2_ layer. These sites are labeled as O1, O2, and O3 in Fig. 6b. Our calculation results reveal that these sites indeed show different vacancy formation energies and suggest that an oxygen vacancy is energetically more favorable in the MnO_2_ layer (O3) (Supplementary Table 1). Further calculations reveal that the formation energy of hydrogen interstitials also depends on the local chemical environment (Supplementary Fig. 13 and Supplementary Note 13). Therefore, the digital superlattice is highly likely to develop certain types of ion ordering after the transfer of ions, leading to a reversible transformation into another crystalline phase instead of phase decomposition.

Our results suggest that the digital superlattices provide a class of materials for electrolytic control of structure and function. The atomically layered structure is found to be critical to enable reversible phase transformation. The underlying mechanism may be correlated to the strength of cation-oxygen bonds arising from the 3*d* versus 5*d* cations as well as the different rotation/distortion of oxygen octahedra. These oxygen octahedral rotations have been predicted by our first-principles calculations to exhibit different magnitudes across the interfaces (Fig. 6a). This digital synthesis approach can be applied to the family of TMOs, which includes but is not limited to iridate and other 3*d* TMOs. Further studies on additional material systems would provide a more comprehensive understanding of the factors governing these structural transformations. These structural transformations can also affect physical properties of the digital superlattices, including emergent phenomena appearing at these oxide interfaces^38^. Therefore, the digital synthesis approach also offers a means to tune emergent functionalities at interfaces through electrolyte-based ion transfer.

In conclusion, we have shown a reversible, electric-field-controlled transformation between distinct phases at RT in atomically layered oxide superlattices, with coincident electronic, optical, and magnetic phase transitions. By contrast, these phenomena are not otherwise observed in the individual constituent oxides, solid solutions or larger period superlattices. Our findings reveal a hitherto-unexplored strategy to develop complex oxides, in which the electrolyte-based ionic control can provide extensive tunability that can be harnessed in electronic/spintronic^6,39^, energy, and environmental applications^19^.

## Methods

### Growth of oxide heterostructures

Thin films and superlattices were grown on atomically flat SrTiO_3_ (STO) (001) substrates by pulsed laser deposition using a KrF excimer laser operating at 248 nm and multiple targets with corresponding chemical stoichiometry. The laser fluence was 0.9 J cm^−2^ and the repetition rate was 1 Hz. The growth temperature and oxygen partial pressure were maintained at 700 °C and 50 mTorr (6.7 Pa). Before growth, STO substrates were prepared in a buffered hydrofluoric acid etch and subsequently annealed at 1000 °C for 3 h to generate atomically flat surfaces as confirmed by atomic force microscopy. During growth, the samples were in situ monitored by using reflection high-energy electron diffraction (RHEED), showing layer-by-layer growth for both La_0.2_Sr_0.8_MnO_3_ and SrIrO_3_. For manganate films and superlattices, RHEED oscillations were observed over the entire deposition process, leading to precise control of interface quality and overall thickness. For iridate and solid solution films, RHEED patterns decay after 5–10 oscillations and the overall thickness was controlled by counting the total number of laser pulses. After growth, the samples were cooled down to room temperature at a rate of ~10 °C per minute in 50 mTorr (6.7 Pa) oxygen.

### In situ and ex situ XRD measurements

XRD measurements were performed by using a high-resolution diffractometer using monochromatic Cu K_α1_ radiation (*λ* = 1.5406 Å). Before ionic liquid gating (ILG), the edges of the samples (5 × 5 mm^2^) were covered with a gold electrode by dc sputtering to form the bottom electrode. Subsequently, conductive gold or platinum wires were connected to the electrode by painting conductive silver adhesive. A thin platinum plate was used as the top electrode. During in situ XRD measurements, the samples were first aligned with the substrate (002) peak without the ionic liquid; then, a small drop of ionic liquid (commercial ionic liquid DEME^+^–TFSI^−^) was added to cover both the film surface and the platinum plate. The voltage between the bottom electrode and the top electrode was initially set to 0 V and then ramped to desired values. Meanwhile, the XRD spectra were collected continuously with a scanning rate of 3° per minute. For ex situ XRD measurements, the samples were gated by ILG in the same configuration. After being transformed into the desired phases, the samples were rinsed several times with acetone and isopropanol to remove the ionic liquid residue before performing XRD scans (such as Fig. 1d). It is noted that the same cleaning procedure was applied for other ex situ measurements.

### Ex situ SIMS measurements

To directly determine the changes in hydrogen and oxygen stoichiometry, we carried out secondary-ion mass spectrometry (SIMS) measurements (using an instrument from IONTOF GmbH). The mass resolution is about 4000 atomic mass units (full-width at half-maximum). During the measurement, the Cesium-ion beam (2 KeV) was rastered over a region of about 250 × 250 μm^2^ but data were collected only in an area of 50 × 50 μm^2^ within that region to avoid disturbance from the crater edge.

### Ex situ polarized neutron reflectivity measurements

To characterize the structural and magnetic depth profile, we performed polarized neutron reflectometry (PNR) on as-grown and gated superlattices by ILG in which 2.5 V was applied for 20 min. We measured the spin-dependent reflectivity as a function of momentum transfer *Q* along the film normal after field cooling to 30 K in an applied magnetic field of 3 T. Because the spin-dependent reflectivity is a function of both the in-plane magnetization and nuclear composition, both the structural and magnetic depth profiles of the superlattices can be reconstructed by fitting the data using the Refl1D software program for *χ*^2^ optimization^40^. Due to the strong perpendicular magnetic anisotropy of the superlattices, it is expected that any component of the net magnetization not aligned along the applied field will instead orient along the film normal, where the neutron scattering selection rules render it invisible. Thus, no in-plane magnetization component is expected to be perpendicular to the applied field, and no spin-flip scattering is expected. We therefore collected only the non-spin-flip reflectivity spectra ↑↑ and ↓↓, where the arrows represent neutrons parallel and antiparallel to the applied field respectively.

### Ex situ X-ray absorption spectroscopy

We carried out X-ray absorption spectroscopy (XAS) measurements at the Mn L-edge and O K-edge at beamlines 4.0.2 and 6.3.1 of the Advanced Light Source at Lawrence Berkeley National Lab. The angle of the incident X-rays is 30 degrees to the sample surface. To probe Mn in the superlattices, we employed two detection modes: total-electron-yield (TEY) mode that is sensitive to the surface and luminescence-yield (LY) mode that probes the entire sample (Supplementary Fig. 7b, c). The two detection modes show similar X-ray absorption spectra, suggesting that the valence changes occur over the entire sample. For the oxygen K-edge, we only employed the TEY mode to avoid the large contributions from the oxide substrates. XAS at the Ir L_3_-edge was measured at beamline 4-ID-D of the Advanced Photon Source at Argonne National Lab. The results were taken by collecting the fluorescence-yield (FY) signal with a grazing incidence geometry.

### In situ transport measurements

Transport measurements were performed in a Quantum Design Dynacool system using a four-probe electrical contact geometry shown in Fig. 4a. The samples were cut into a rectangular shape (2 × 5 mm^2^). Patterned electrodes (AuPd, ~ 50 nm) were deposited on the surface of the superlattice by dc sputtering. Then a thin Pt foil was used as the top electrode. Bias voltage (V_g_) was applied between the top electrode and the superlattice, contacted by a drop of ionic liquid (DEME^+^-TFSI^−^). The source-drain current was set to be 2 µA for all measurements. To measure magnetoresistance, we applied magnetic fields of up to 7 T in the out-of-plane direction. The transport measurements were performed under vacuum in the cryostat (Dynacool system chamber).

### Ex situ optical transmittance measurements

We used superlattices grown on double-side polished STO (001) substrates (10 × 10 mm^2^) for optical transmittance measurements. A bare double-side polished STO (001) substrate was processed through the same thermal cycling as the other samples and was used as a reference for the optical transmittance measurements. Ex situ optical transmittance spectra were taken in air at room-temperature with spectrophotometers (Agilent Cary 6000i UV/Vis/NIR), which cover the visible and near-infrared range with wavelengths between 400 nm and 1800 nm.

### In situ and ex situ magnetic measurements

In situ magneto-optic Kerr effect (MOKE) measurements were performed by using a lateral top gate electrode as shown in Supplementary Fig. 11a. The gate electrode was deposited on an insulating LaAlO_3_ (LAO) single crystal, which was mounted next to the superlattice on an insulating polymer platform. The measurements were carried out in vacuum in a cryostat. Liquid nitrogen was used to cool down the superlattice.

To obtain quantitative results over a wider temperature range, a Quantum Design 7T-SQUID magnetometer was employed to perform ex situ magnetic measurements. The magnetic hysteresis loops were measured at 10 K after field cooling in 7 T. The temperature dependent magnetization was measured during the warming process with an applied field of 0.2 T. Magnetic properties were measured both along the out-of-plane (Fig. 5) and in-plane ([100], Supplementary Fig. 12) directions.

### First-principles calculations

We performed non-collinear spin-resolved density-functional-theory (DFT) calculations using the Vienna Ab initio Simulation Package (VASP)^41,42^ to estimate the defect formation energy of an oxygen vacancy or a hydrogen interstitial. The PBEsol functional^43^ was used in the form of the projector augmented wave method^44^. DFT + U approach developed by Dudarev et al.^45^ was adopted to describe the correlation effects of the system. Additional information about our DFT calculations is included in the Supplementary Note 13.

For simplicity, we neglected the small amount of La doping in the superlattices and used the SrIrO_3_/SrMnO_3_ (SIO/SMO) supercell in the calculation, which still reveals the role of B-site cation ordering. The supercell is composed of alternating one-unit-cell of SIO and SMO stacking in the out-of-plane direction while the in-plane direction contained double perovskite cells to account for octahedral rotation, resulting in a total chemical formula of Sr_4_Ir_2_Mn_2_O_12_. Subsequently an oxygen vacancy or a hydrogen interstitial was introduced into the superlattice (see Supplementary Note 13 for more details). All structures were relaxed until the electronic convergence of 10^−6^ eV was reached and the force on each atom was smaller than 0.01 eV Å^−1^. The energy cutoff of the plane-wave basis was set as 550 eV, and the 6 × 6 × 4 Gamma-centered Monkhorst-Pack grid was employed in the calculations.

## Supplementary information

Supplementary Information

## Data Availability

The data that support the findings of this study are available from the corresponding authors upon reasonable request.
