# Supplementary material for: Emergent electric field control of phase transformation in oxide superlattices
Source: Nat Commun. 2020 Feb 14;11:902. doi: 10.1038/s41467-020-14631-3 (PMC7021769; doi:10.1038/s41467-020-14631-3)
Supplement: Supplementary file 1 — Supplementary Information [file 41467_2020_14631_MOESM1_ESM.pdf]

Supplementary Information

Emergent electric field control of phase transformation in oxide superlattices

Yi et al.

Supplementary Note 1: RHEED oscillations and patterns

Supplementary Figure 1 shows the reflection high-energy electron diffraction (RHEED) oscillations for the $[(La_{0.2}Sr_{0.8}MnO_3)_1\ (SrIrO_3)_1]_n$ superlattices. Oscillations for both $La_{0.2}Sr_{0.8}MnO_3$ and $SrIrO_3$ sublayers are clearly observed during the entire deposition process, revealing the layer-by-layer growth of both materials. Insets shows the RHEED patterns of the $SrTiO_3$ (STO) substrate, the sample when 5 repetitions were deposited (n=5) and the final sample with 20 repetitions (n=20), all of which indicate smooth surfaces. The surface roughness of the final surface of the sample is likely to slightly increase as compared to that of the substrate.

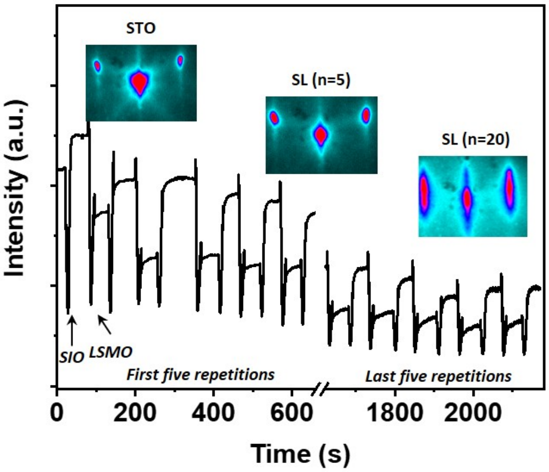

Supplementary Figure 1 | RHEED oscillations and patterns for the growth of $[(La_{0.2}Sr_{0.8}MnO_3)_1\ (SrIrO_3)_1]_n$. RHEED oscillations for the first and last five repetitions are shown. One repetition consists of one-unit-cell of LSMO and SIO. Insets show the RHEED patterns of the $SrTiO_3$ substrate, the superlattice consisting of five repetitions (n=5) and the final superlattice that consists of twenty repetitions (n=20).

Supplementary Note 2: XRD results on the four types of oxides

Supplementary Figure 2(a) shows the XRD spectra of as-grown superlattices (SL) and solid solution (SS) films. For the rest of this paper, the term superlattice is used to refer to $[(La_{0.2}Sr_{0.8}MnO_3)_1(SrIrO_3)_1]_{20}$ superlattice unless otherwise stated. The fundamental (002) Bragg peaks are similar. The superlattices show pronounced satellite peak due to the ordering of B-site transition metal cations, which is absent in the solid solution films with a random distribution of B-site cations.

Supplementary Figure 2(b) to (f) show the in-situ XRD results of superlattices (SL), solid solution (SS) films, $La_{0.7}Sr_{0.3}MnO_3$ films, $La_{0.2}Sr_{0.8}MnO_3$ films and $SrIrO_3$ films under the same gating procedure.

We first applied incremental positive voltages (from 0 V to +2.5 V). After ramping to each desired value, the voltage was held for ~ 12 minutes at each step. Finally, negative -3 V was applied for ~30 minutes to check reversibility. As show in Supplementary Figure 2(b) to (f), we found that only the superlattices show a reversible structural transformation with ~7% lattice change. The (002) peak of all other samples disappears below 2 V and cannot be reversed by applying -3 V, indicating phase decomposition. For a clear demonstration, we only show the results below 2.0 V for $SrIrO_3$ films, which exhibit an abrupt decay of the (002) peak above 2.0 V that cannot be reversed by applying -3 V. It is noted that we did not observe a clear tri-state phase transformation as what has been reported in $H_xSrCoO_{3-\delta}$[1], although the transfer of both hydrogen and oxygen ions was confirmed in our superlattices. Further electrochemical studies are required to fully understand the kinetic process of the dual-ion transfer in our superlattices.

It is noted that the in-situ XRD measurements under ILG were carried out in air at room temperature. However, similar structural transformations (based on ex-situ XRD measurements) were also realized in an air-free environment in the presence of 10^{-3} Torr (0.13 Pa) of argon, suggesting that the gaseous environment does not have significant effects on the electric-field-controlled phase transformation in the superlattices.

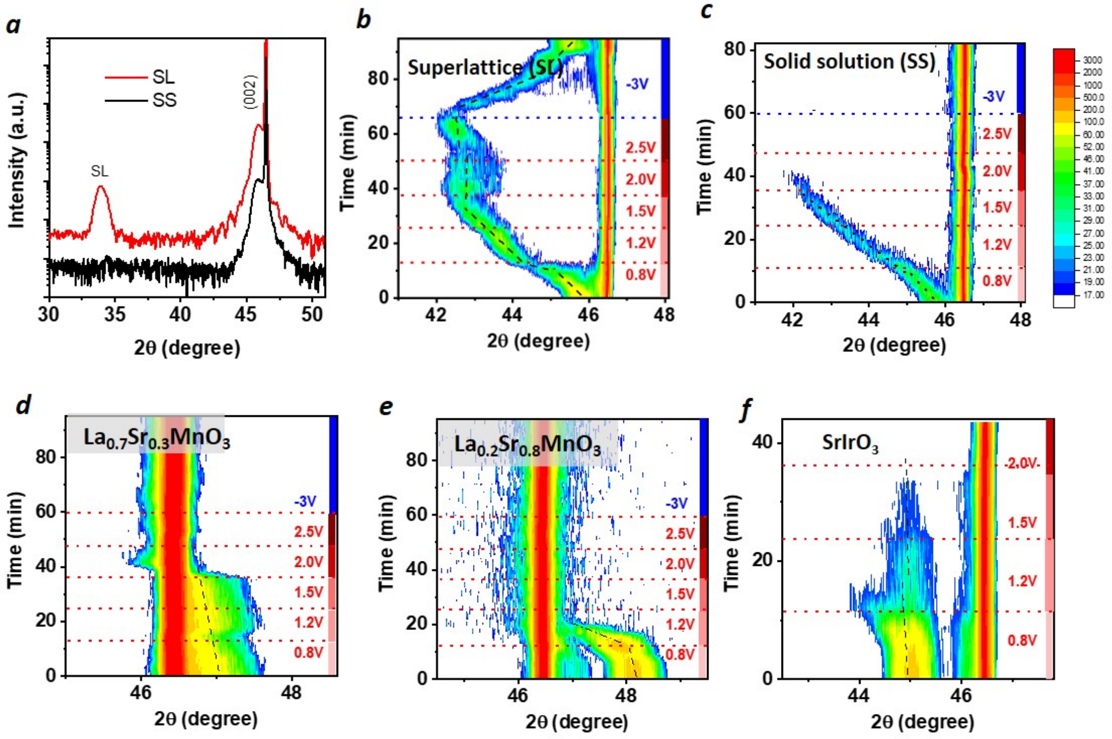

Supplementary Figure 2 | In-situ XRD results of four types of samples during voltage cycling. (a) θ-2θ XRD spectra of as-grown superlattices (SL) and solid solution (SS) films. (b-f) In-situ XRD results

during voltage cycling (0.8 V, 1.2 V, 1.5 V, 2.0 V, 2.5 V and -3 V) for (b) superlattices, (c) solid solution films, (d) $La_{0.7}Sr_{0.3}MnO_3$ films, (e) $La_{0.2}Sr_{0.8}MnO_3$ films and (f) $SrIrO_3$ films.

Supplementary Note 3: XRD results on superlattices with larger periods

Supplementary Figure 3(a) shows the XRD spectra of as-grown superlattices with larger periods, i.e. $[(La_{0.2}Sr_{0.8}MnO_3)_2(SrIrO_3)_2]_{10}$ (m=2) and $[(La_{0.2}Sr_{0.8}MnO_3)_4(SrIrO_3)_4]_5$ (m=4). The total thickness is approximately 16 nm (40 unit-cells). Supplementary Figure 3(b) and 3(c) show the in-situ XRD results of superlattices with m=2 and m=4 under the same gating procedure in Supplementary Note 2. For the m=2 sample, the lattice expansion is about ~5.5 % upon application of positive voltages. However, the (002) peak disappears at around 1.5V and the change is irreversible. For the m=4 sample, the lattice expansion is ~2 % before the disappearance of the (002) peak. It is noted that the peak around 43.6° in Supplementary Figure 3(c) is the superlattice satellite peak. The results reveal that as the period of the superlattices increases, the magnitude of the lattice change decreases and the change becomes irreversible. In other words, the superlattices with larger periods behave like the single-phase parent oxide films shown in Supplementary Figure 2.

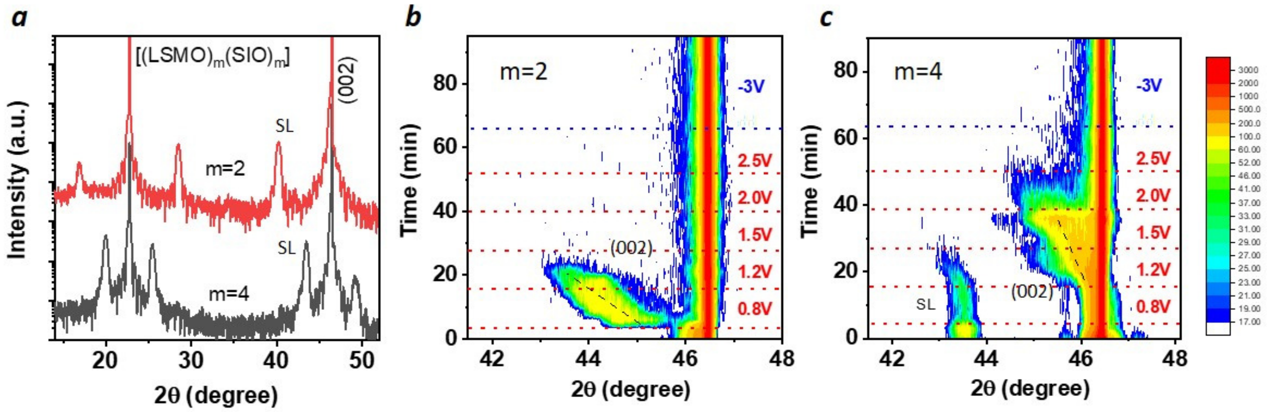

Supplementary Figure 3 | In-situ XRD results of superlattices with larger periods during voltage cycling. (a) θ-2θ XRD spectra of as-grown superlattices $[(La_{0.2}Sr_{0.8}MnO_3)_2(SrIrO_3)_2]_{10}$ and $[(La_{0.2}Sr_{0.8}MnO_3)_4(SrIrO_3)_4]_5$. (b-c) In-situ XRD results during voltage cycling (0.8 V, 1.2 V, 1.5 V, 2.0 V, 2.5 V and -3 V) for (b) $[(La_{0.2}Sr_{0.8}MnO_3)_2(SrIrO_3)_2]_{10}$ and (c) $[(La_{0.2}Sr_{0.8}MnO_3)_4(SrIrO_3)_4]_5$. It is noted that the peak around 43.6° in (c) is the superlattice satellite peak.

Supplementary Note 4: Evolution and SIMS results of phase B under different conditions

Supplementary Figure 4(a) shows stability of the ionic liquid gating (ILG) induced phase B under ambient conditions. The superlattice was first stabilized in phase B under ILG. Then ionic liquid residue

was removed. The phase B sample was kept under ambient conditions while θ-2θ XRD spectra were taken after two hours, one day, two days and four days. The (002) peak shows a slow and gradual change towards the higher angle, indicating a slow transition to phase A. The results indicate that ILG induced phase B is metastable under ambient conditions. By contrast, the application of -3 V can quickly restore phase A (blue).

Supplementary Figure 4(b) shows stability of the ILG induced phase B under thermal annealing process. The superlattice was first stabilized in phase B by ILG (red). Then the phase B sample was thermally annealed at 350 °C for 15 minutes in oxygen gas (~ 100 Torr). The θ-2θ XRD spectra (black) show that the (002) peak shifts to a higher angle, indicating that thermal annealing in oxygen gas induces a quick transformation to phase A, which is likely due to incorporation of oxygen ions and loss of hydrogen ions.

Although it is challenging to directly probe oxygen vacancies, SIMS results in Supplementary Figure 4(c) allow us to infer the presence of oxygen vacancies. Firstly, we stabilized two superlattices in phases A and two superlattices in phase B under ILG. Next, we thermally annealed one phase A sample and one phase B sample in isotope $^{18}O_2$ gas at 350 °C for 15 minutes. Then the depth profiles of ^{18}O signal of these four samples were measured by using SIMS, as shown in Supplementary Figure 4(c). We find that no excess ^{18}O above its natural level is observed in superlattices under ILG. The slightly lower ^{18}O signal in phase B may be correlated to the extraction of oxygen anions in phase B under ILG. On the other hand, the difference is significant for samples after thermal annealing. The phase B sample after annealing shows a dramatic increase in ^{18}O signal over the entire sample, while the phase A sample after annealing only shows increase at the surface region.

The depth profile of ^{18}O after thermal annealing is determined by both the surface exchange ($^{16}O/^{18}O$) coefficient and the bulk oxygen diffusion and has been discussed in many studies[2]. Although the depth profile of ^{18}O does not directly measure the oxygen vacancy concentration, the dramatic difference between the depth profiles of the phase A and B samples after annealing indicates that the phase B sample has a much higher oxygen diffusion coefficient than phase A. Therefore, the larger amount of ^{18}O in the bulk of the thermally annealed phase B sample indicates a higher concentration of oxygen vacancies in the bulk of the sample, while the ^{18}O at the surface of both phase A and B samples are a manifestation of the ^{18}O surface exchange process.

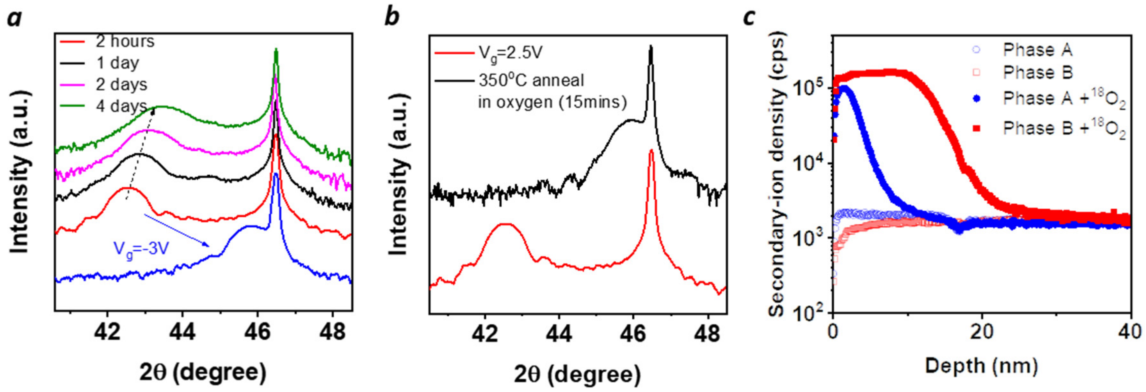

Supplementary Figure 4 | Stability of the phase B and chemical characterization. (a) Ex-situ XRD results of phase B under ambient conditions, taken at different time intervals. By contrast, the application of -3 V can quickly restore phase A (blue) from phase B (red). (b) Ex-situ XRD results of the sample in phase B after application of 2.5 V (red) and then under thermal annealing in oxygen gas (black). (c) Depth profiles of ^{18}O in four types of superlattices. The SIMS results from "phase A" and "phase B" samples (hollow symbols) were measured on superlattices that were stabilized by ILG. The SIMS results from "phase A+$^{18}O_2$" and "phase B+$^{18}O_2$" samples (solid symbols) were measured on superlattices that were first stabilized into phase A and B by ILG and then thermally annealed in isotope $^{18}O_2$ gas at 350 °C. As discussed in Supplementary Note 4, the results suggest the creation of oxygen vacancies in phase B.

Supplementary Note 5: Reciprocal space map of superlattices

Supplementary Figure 5 shows the Reciprocal space mapping (RSM) of superlattices in both phases in the (103) crystalline plane of STO substrate. The results reveal that both phases of the superlattices are coherently strained to the underlying STO substrate with identical in-plane lattices, while a significant modulation of out-of-plane lattices are induced in the superlattices.

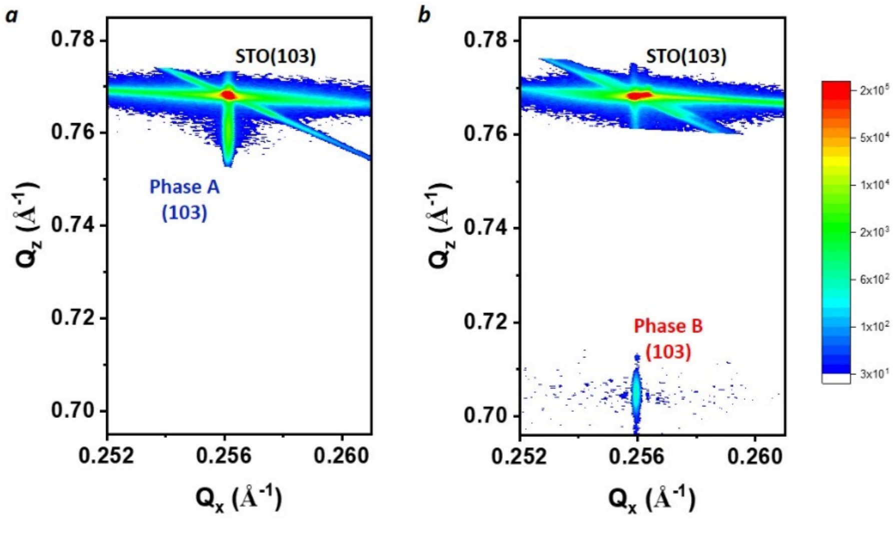

Supplementary Figure 5 | Reciprocal space mapping (RSM) of superlattices in two phases. RSM of superlattices in phase A (a) and phase B (b) in the (103) crystalline plane of the STO substrate. $Å^{-1}$

represents the reciprocal space units, and Q_x and Q_z represent projected directions along [100] and [001] directions in reciprocal space.

Supplementary Note 6: Polarized neutron reflectivity

We measured two samples by polarized neutron reflectivity (PNR): an as-grown superlattice (phase A) and a gated superlattice that was stabilized in phase B by ILG (2.5 V for 20 mins). For this experiment, the phase B sample had been kept at ambient condition for a few days before being measured for PNR. We noted that the gated sample had shown partial relaxation to phase A, as indicated by XRD (Supplementary Figure 4(a)) and by resistivity measurements. Nevertheless, the differences between the two samples still reveal evidence that is consistent with the chemical (Figure 2) and magnetic (Figure 5) results.

The as-grown and gated samples yield the depth profiles shown in Supplementary Figure 6(b) and 6(d). We first study the nuclear scattering length density (SLD), which reveals results that are consistent with structural characterization in Figure 1 and chemical characterization in Figure 2. The best fit nuclear SLD of the bulk (interior region) superlattice is reduced from 4.6×10^{-4} nm^{-2} to 4.15×10^{-4} nm^{-2}, while the surface nuclear SLD is even more significantly reduced from 3.98×10^{-4} nm^{-2} to 2.29×10^{-4} nm^{-2}. The huge differences (-0.45×10^{-4} nm^{-2} near the substrate and -1.69×10^{-4} nm^{-2} near the surface) are far in excess of those achieved by using a Gd capping layer to extract oxygen in either (La,Sr)MnO_3 and (La,Sr)CoO_3, where changes of approximately 0.1×10^{-4} nm^{-2} were observed[3,4]. Therefore, the results indicate a high concentration of oxygen vacancies and likely an incorporation of hydrogen ions. It is noted that the scattering length of hydrogen is large and negative with a value of -3.741 *fm* while both $SrIrO_3$ and (La,Sr)MnO_3 have net positive nuclear scattering lengths. Therefore, hydrogen incorporation is expected to greatly suppress the nuclear SLD. We also note that change of nuclear SLD is more significant at the surface, which is consistent with results in Figure 2. The partial relaxation of phase B might also contribute to the difference between the surface and interior regions in Supplementary Figure 6.

Next, we study the magnetic SLD. The data of the as-grown sample yields a uniform magnetic depth profile except for a small magnetic dead layer at the surface (Supplementary Figure 6(b)). This dead layer is coincident with a region of slightly reduced nuclear SLD, as also seen in recent examples of PNR on (La,Sr)MnO_3 and (La,Sr)CoO_3 surfaces[3,4]. Comparing the as-grown sample to the gated sample reveals immediate differences. In particular, the splitting between the ↑↑ and ↓↓ reflectivity (Supplementary Figure 6(a) and (c)) is largely reduced. Fitting the reflectivity of the gated sample (Supplementary Figure

6(d)) reveals an approximately three-fold reduction in the magnetic SLD, which is directly proportional to the magnetization. This is also consistent with the suppression of ferromagnetism in Figure 5.

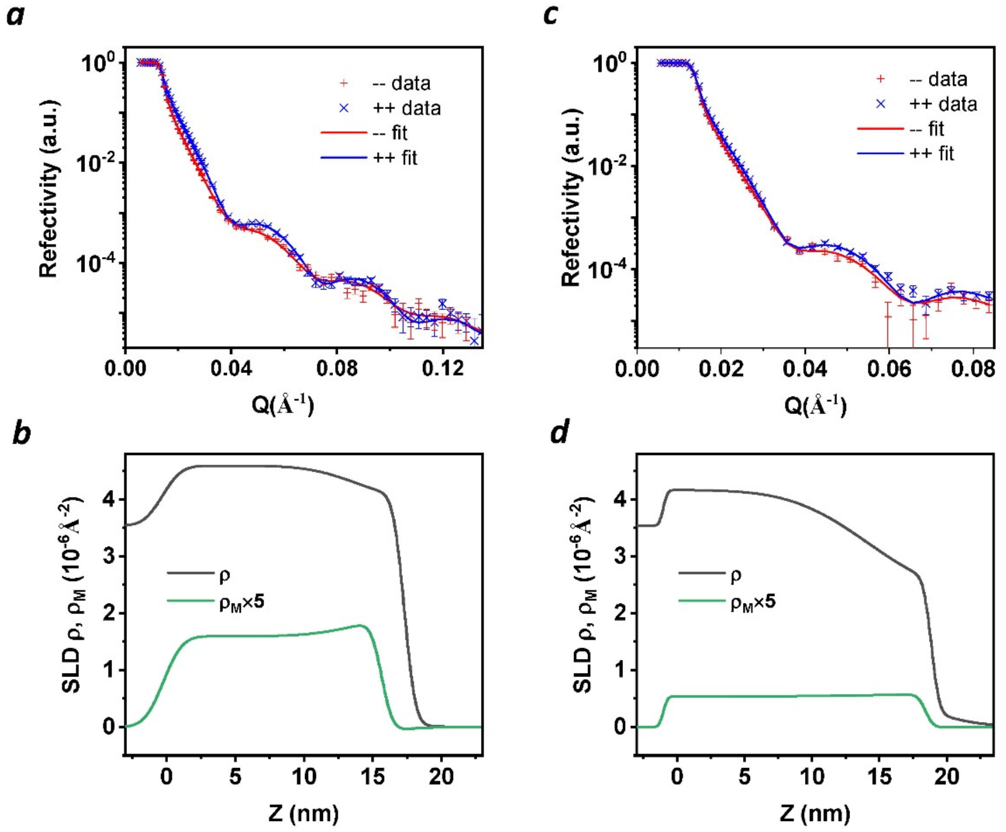

Supplementary Figure 6 | Polarized neutron reflectivity results of the superlattices. (a) Fitted spin-dependent polarized neutron reflectivity of phase A and (b) the nuclear (black) and magnetic (green) SLD profiles deduced from the fits of phase A. (c) Fitted spin-dependent polarized neutron reflectivity of a positively gated superlattice in phase B and (d) the associated nuclear (black) and magnetic (green) SLD profiles. Error bars represent ±1 standard deviation.

Supplementary Note 7: Additional XAS measurements of superlattice

Supplementary Figure 7(a) shows the X-ray absorption (XA) spectra at the Mn L-edge of the superlattice for the initial voltage cycling. The XA spectra was taken ex-situ in the total-electron-yield (TEY) mode. Although a small difference was observed between the as-grown pristine state and the reversibly gated state, the difference is small when compared to the valence change between the two phases of superlattice. The results show that the modulation is reversible, consistent with the structure change.

The Mn XA spectra taken in TEY mode mainly probe the surface 5~10 nm. To probe the entire superlattice, we also used luminescence-yield (LY) to collect XA signal. Supplementary Figure 7(b) shows the schematic diagram of XA measurements using LY mode, wherein LY signal from the STO substrate is detected by a photodiode. At the absorption edge (Mn L-edge), the intensity of x-rays entering the STO substrate decreases due to absorption by the films, leading to a decrease of LY signal. Therefore, the XA spectra obtained in LY mode probe the entire film thickness. Supplementary Figure 7(c) shows luminescence signal at the Mn L-edge for the same voltage cycling, as the TEY signal in Supplementary Figure 7(a). Therefore, valence change and ion transfer occur over the entire film thickness, consistent with the SIMS measurements.

Supplementary Figure 7(d) shows the XA spectra at the Ir L_3-edge, taken in fluorescence-yield (FY) mode which also probes the entire sample. The shift of absorption peak to lower energy by ~0.8 eV and the reduction of peak intensity in phase B indicate a decrease of Ir oxidation state by ~1[5,6].

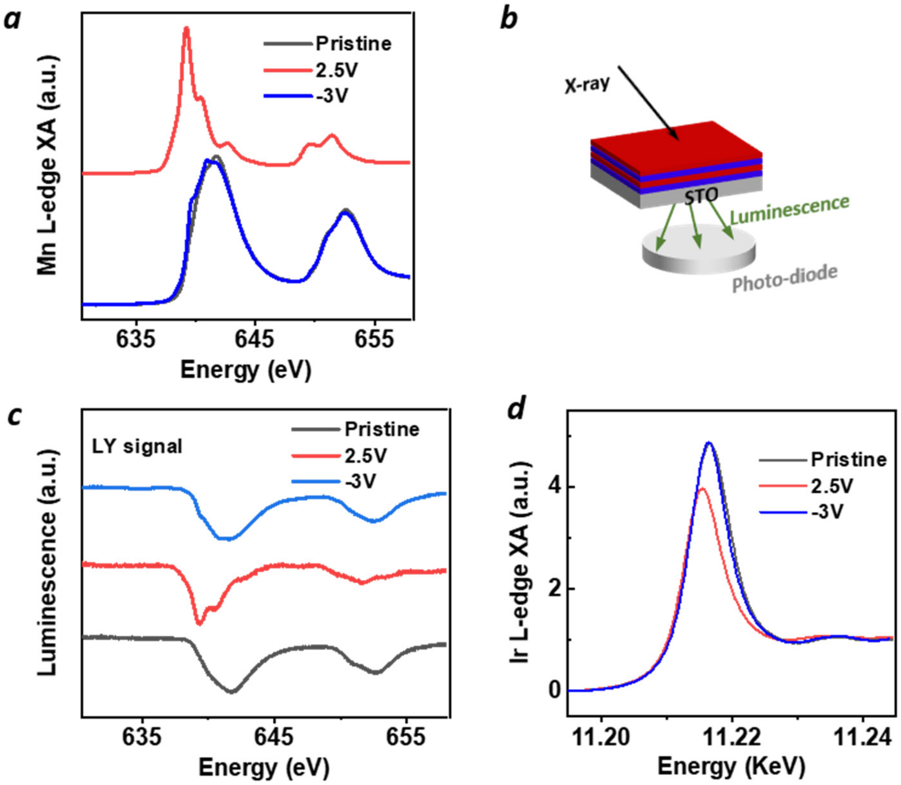

Supplementary Figure 7│XAS measurements to check reversibility and depth dependence. (a) X-ray absorption (XA) spectra at the Mn L-edge (taken in TEY mode) in initial voltage cycling. (b) Schematic of X-ray absorption spectroscopy (XAS) measurements by using the luminance-yield (LY) mode. (c) Luminescence signal at the Mn L-edge in the same voltage cycling. (d) XAS at the Ir L_3-edge (taken in FY mode) of the superlattices in initial voltage cycling.

Supplementary Note 8: Fitting Mn oxidation states of superlattices

Supplementary Figure 8(a) shows the fitting of XA spectrum of phase A (blue) by using the linear superposition of two reference spectra, i.e. $LaMnO_3$ films (close to Mn^{3+}) and $CaMnO_{3-\delta}$ films (close to Mn^{4+}). The best fitting curve (black dash line) indicates that the Mn oxidation state is close to +3.5, consistent with previous studies[7].

Supplementary Figure 8(b) shows the XA spectra of phase B (red) and a reference sample MnF_2 (Mn^{2+}). The similar multiplet features and peak positions show that the Mn oxidation state of phase B is close to +2.

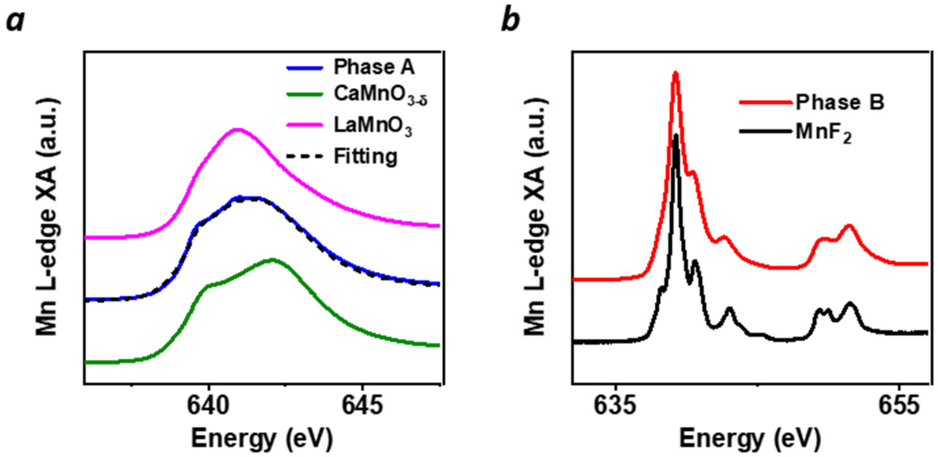

Supplementary Figure 8 | Comparing the Mn-L edge XAS of superlattices to references. (a) XA spectra at Mn L-edge (taken in TEY mode) of the superlattice in phase A (blue), $LaMnO_3$ films (close to Mn^{3+}) and $CaMnO_{3-\delta}$ (close to Mn^{4+}). The fitted spectrum (black dash line) is a linear superposition of XA spectra of Mn^{3+} and Mn^{4+}. (b) XA spectra at Mn L-edge (taken in TEY mode) of the superlattice in phase B (red) and a reference sample MnF_2 (Mn^{2+}) (black).

Supplementary Note 9: Valence changes of singe-phase oxide films under ILG

Supplementary Figure 2 reveals that all the single-phase oxide films, i.e. $La_{0.7}Sr_{0.3}MnO_3$ films, $La_{0.2}Sr_{0.8}MnO_3$ films and $SrIrO_3$ films, show irreversible structural changes under ILG. Here we also measured the XA spectra of $La_{0.2}Sr_{0.8}MnO_3$ films and $SrIrO_3$ films after irreversible structural changes were induced by ILG. Supplementary Figure 9 shows the XA spectra of these films in the as-grown state (black) and in the positively gated state (red). For the gated state by ILG, the voltage of +2.5 V was applied for 20 minutes, which leads to the diminishing of (002) Bragg peak (Supplementary Figure 2).

Supplementary Figure 9(a) and 9(b) show the Mn L-edge and oxygen K-edge XA spectra of $La_{0.2}Sr_{0.8}MnO_3$ films. For the gated state (red), the XA spectrum at Mn L-edge shows an oxidation state

close to +3 (Supplementary Figure 9(a)). Moreover, the spectral features at the oxygen K-edge associated with O-Mn hybridizations are not fully suppressed in the gated state (Supplementary Figure 9(b)). By comparing the XA spectra of $La_{0.2}Sr_{0.8}MnO_3$ films to that of the superlattices (Figure 3(a) and 3(c)), the differences reveal a much smaller valence change in $La_{0.2}Sr_{0.8}MnO_3$ films, suggesting a smaller magnitude of ion transfer despite irreversible structural changes that have been induced in $La_{0.2}Sr_{0.8}MnO_3$ films. We note that this is consistent with the smaller lattice change (~ 2 %) in $La_{0.2}Sr_{0.8}MnO_3$ films, revealed by in-situ XRD under ILG.

Supplementary Figure 9(c) shows the oxygen K-edge XA spectra of $SrIrO_3$ films. The spectral features associated with O-Ir hybridization are fully suppressed in the gated state, revealing a significant valence change. Therefore, it is likely that ion transfer also occurs in $SrIrO_3$ films under ILG. However, the lattice framework of $SrIrO_3$ decomposes quickly as shown in Supplementary Figure 2(f). One possible origin is that the perovskite phase of $SrIrO_3$ is metastable in the bulk and only stabilized by epitaxial strain on STO substrates.

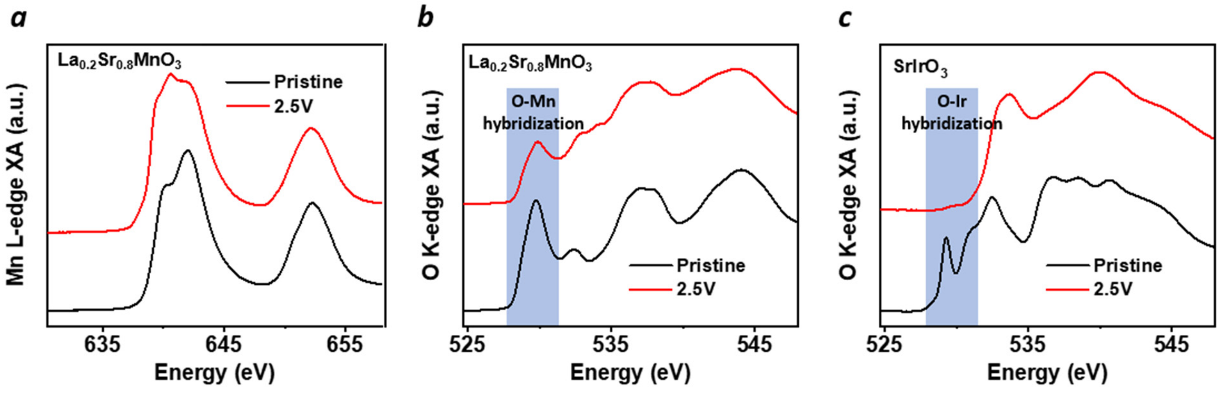

Supplementary Figure 9 │ XAS of singe-phase oxides films under ILG. (a,b) XA spectra at (a) Mn L-edge and (b) oxygen K-edge for $La_{0.2}Sr_{0.8}MnO_3$ films in pristine state (black) and in the positively gated state by ILG with the application of +2.5V (red). (c) XA spectra at oxygen K-edge for $SrIrO_3$ films in the pristine state (black) and in the positively gated state by ILG with the application of +2.5V (red).

Supplementary Note 10: Additional transport measurements

In-situ transport measurements under ILG were performed by using the device shown in Figure 4(a). Supplementary Figure 10(a) shows the resistivity as a function of time when different positive bias voltages (V_g from 0.8 V to 2.5 V) were applied to the low-resistance state (phase A) at RT. Supplementary Figure 10(b) shows the resistivity as a function of time for phase B (high-resistance state) under different conditions at RT. The superlattice was first induced into phase B (red) by applying +2.5 V. Then temporal

changes were measured for phase B samples with zero bias voltage in air (green), under vacuum in the cryostat (PPMS chamber, orange) and with the application of -3 V in PPMS chamber (blue). When the voltage was set to 0 V, the resistance was measured for a few hours and showed small changes. These changes are consistent with structural results in Supplementary Figure 4(a). In addition, we did not observe a significant difference between the phase B sample in air and under vacuum (PPMS chamber). Supplementary Figure 10(c) shows that the modulation of resistivity in the superlattices is highly reversible by applying repeated cycling of V_g=2.5 V and V_g=-3 V.

Supplementary Figure 10(d) shows magnetoresistance (blue) and magnetic (red) hysteresis loops at 40 K of an as-grown superlattice. Magnetic field was applied in the out-of-plane direction. Therefore, we can use magnetoresistance to infer the magnetic properties. Supplementary Figure 10(e) shows the magnetoresistance hysteresis loops of the superlattice at 40 K in different gated states, indicating a full suppression of ferromagnetism by applying the positive voltage. Moreover, this suppression can be reversed by applying -3 V (blue). These magnetoresistance measurements are consistent with the magnetic results in Figure 5.

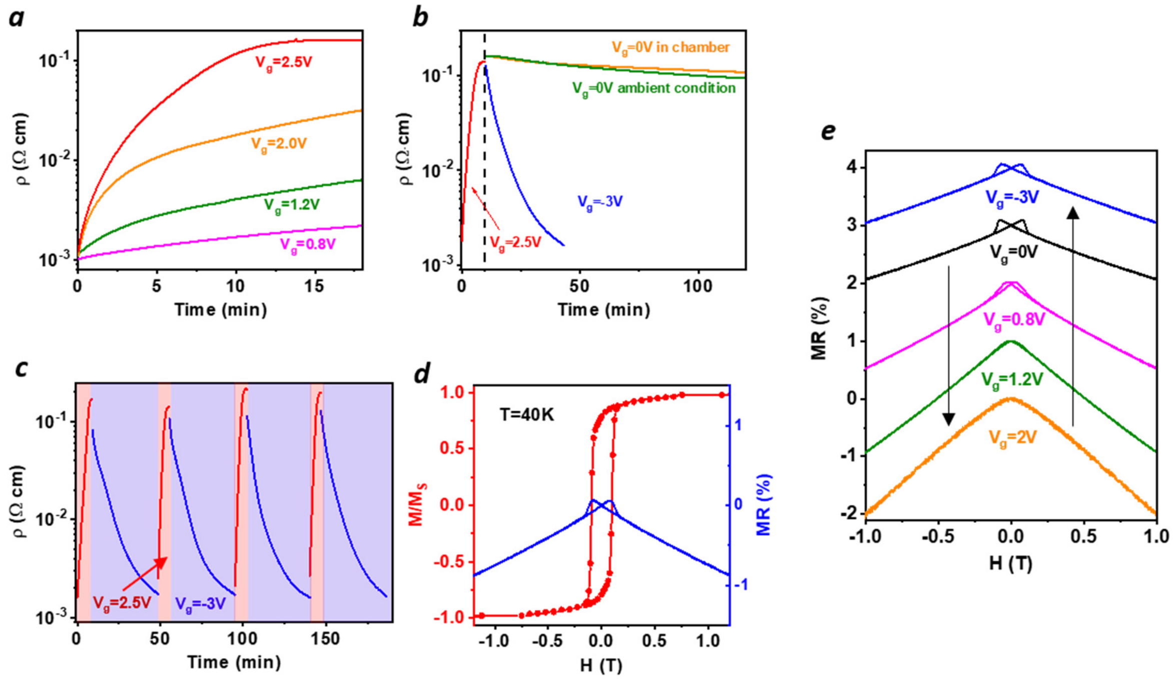

Supplementary Figure 10 | Further transport measurements of superlattices under ILG. (a) Resistivity as a function of time with the application of positive voltages (V_g from 0.8 V to 2.5 V) to the low-resistance state (phase A) at RT. (b) Resistivity as a function of time for phase B (high-resistance state) under different conditions at RT. Temporal changes were measured, for phase B sample with zero bias voltage in air (green), in the PPMS chamber (orange) and with the application of -3 V in the PPMS chamber (blue). (c) Temporal changes of resistivity during repeated cycling of V_g=2.5 V and V_g=-3 V. (d)

Comparison of the magnetoresistance (blue) and magnetic (red) hysteresis loops at 40 K of an as-grown superlattice. Magnetic field was applied in the out-of-plane direction for both measurements. (e) Magnetoresistance hysteresis loops of the superlattice at 40 K in different gated states. An offset of 1 % is applied between loops for illustration.

Supplementary Note 11: Additional in-situ MOKE measurements

Supplementary Figure 11(a) shows the schematic diagram of the device for the in-situ magneto-optic Kerr effect (MOKE) measurements. The gate electrode was deposited on an insulating $LaAlO_3$ (LAO) single crystal, located next to the superlattice. Ionic liquid was placed to cover the gate electrode and the superlattice. MOKE signals were detected with the presence of ionic liquid and mainly probed the out-of-plane magnetization in the superlattices under different gate voltages.

Supplementary Figure 11(b) shows the temperature dependence of the MOKE signals when different V_g were applied. The results are consistent with ex-situ magnetic measurements in Figure 5(c), revealing a decrease of T_c with increasing V_g. The ferromagnetism is fully suppressed to below the lowest measuring temperature of ~80 K (in our liquid nitrogen cooled setup) when V_g=2 V was applied.

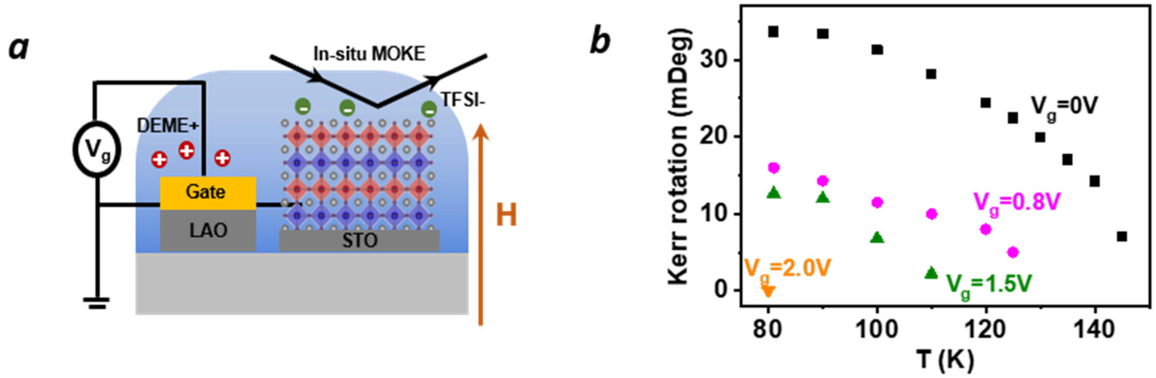

Supplementary Figure 11 | In-situ magnetic measurements under ILG. (a) Schematic diagram of the device for the in-situ magneto-optic Kerr effect (MOKE) measurements. (b) Temperature dependence of the MOKE signals (down to 80 K) when different V_g were applied.

Supplementary Note 12: Additional ex-situ SQUID measurements

Supplementary Figure 12(a) shows the magnetic hysteresis loops along the out-of-plane direction during repeated cycling (first and fifth voltage cycling of +2.5 V and -3 V). The results show that the magnetic modulation is highly reversible. It is noted that we observed a small difference between the

superlattices in the as-grown state and in the reversibly gated state in the initial voltage cycling (Figure 5(b)), which is not clear during repeated cycling as shown in Supplementary Figure 12(a).

To fully understand the change of ferromagnetism, we also measured the in-plane magnetic hysteresis loops along the [100] direction, which are complementary to the results in Figure 5(b). The results are shown in Supplementary Figure 12(b). By increasing positive V_g, both saturation moment M_S and remnant moment M_R along the in-plane direction decrease. The combination of results in Supplementary Figure 12(b) and Figure 5(b) indicates a full suppression of ferromagnetism in phase B of the superlattices and not just a change in magnetic anisotropy.

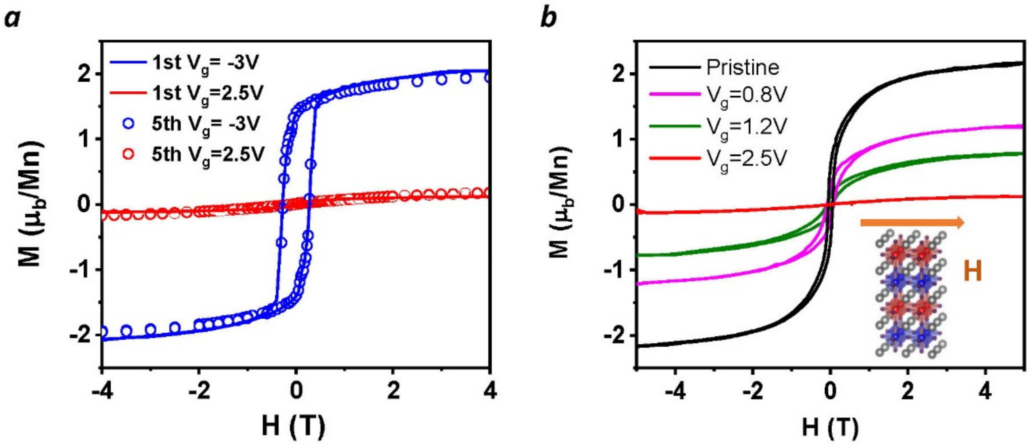

Supplementary Figure 12 | Ex-situ magnetic measurements of superlattices under ILG. (a) Magnetic hysteresis loops along the out-of-plane direction during repeated cycling, showing that the magnetic modulation is highly reversible. (b) In-plane magnetic hysteresis loops at different gated states. We measured in-plane magnetic hysteresis loops along the [100] direction.

Supplementary Note 13: First-principles calculations of the point-defect formation energies

We performed non-collinear spin-resolved density-functional-theory (DFT)[8,9] calculations using the Vienna Ab initio Simulation Package (VASP)[10,11] to estimate the defect formation energy of an oxygen vacancy or a hydrogen interstitial. PBEsol functional[12] was used in the form of the projector augmented-wave[13] method. $4s^2 4p^6 5s^2$ electrons of Sr, $5d^7 6s^2$ of Ir, and $3p^6 3d^5 4s^2$ of Mn were treated as valence states. DFT+U approach developed by Dudarev et al[14] was adopted to describe the correlation effects of the system, with U_{eff} set as 3.0 eV for *d* orbitals of Mn[15] and 2.0 eV for those of Ir[16].

As shown in Figure 6(a), the supercell is composed of alternating one perovskite cell of $SrIrO_3$ and $SrMnO_3$ stacking in the out-of-plane direction while the in-plane direction contained double perovskite

cells to account for octahedral rotation, resulting in a total chemical formula of $Sr_4Ir_2Mn_2O_{12}$. The in-plane lattice parameter was fixed as that of $SrTiO_3$ under the PBEsol+U condition[17,18], while the average *c/a* ratio of the superlattice without defects was optimized to be 1.015. The rotation patterns of IrO_6 and MnO_6 octahedra along the **c** axis were found to be out-of-phase with the angles being 14.5° and 2.9°, respectively, as shown in Figure 6(a). This result is consistent with previous results[19] which incorporated a different in-plane lattice constant and optimization process, thus suggesting the robustness of our calculations.

Subsequently an oxygen vacancy or a hydrogen interstitial was introduced into the supercell. More specifically, three types of oxygen vacancy positions were tested, i.e., in the SrO layer, in the IrO_2 layer, or in the MnO_2 layer (corresponding to O1, O2, and O3, respectively, as illustrated in Figure 6(b)). As for the hydrogen interstitial, 18 initial positions were generated by Voronoi analysis[20], as shown in Supplementary Figure 13(a). All structures were relaxed until the electronic convergence of 10^{-6} eV was reached and the force on each atom was smaller than 0.01 eV $Å^{-1}$. The energy cut-off of the plane-wave basis was set as 550 eV and the 6×6×4 Gamma-centered Monkhorst-Pack grid was employed in the calculations.

The defect formation energy (DFE), E^f, was calculated by using equation (1)[21]:

$$E^f = E_{DEF} - E_0 - n\mu + q(\varepsilon_F + V_{corr}) \quad (1)$$

where E_{DEF} is the energy of the supercell with the defect; E_0 is the energy of the supercell without defect; n is the number of atoms being removed (<0) or added (>0); μ is the chemical potential of the corresponding atoms; q is the number of electrons removed (>0) or added (<0) associated with the defect; ε_F is the Fermi energy of the system without defect; V_{corr} is a correction term originating from the electrostatic potential misalignment between the system with and without the defect.

Here we only consider charge-neutral defects. The first two terms can be directly obtained from our DFT calculations as discussed above. The exact value of the chemical potential μ is often hard to determine. Instead, we estimate the upper/lower bound of μ. The following thermodynamics constrains limit the chemical potential for each element as:

$$\mu_{Sr}[superlattice] < \mu_{Sr}[metal] \quad (2)$$

$$\mu_{Ir}[superlattice] < \mu_{Ir}[metal] \quad (3)$$

$$\mu_{Mn}[superlattice] < \mu_{Mn}[metal] \quad (4)$$

$$\mu_O[superlattice] < \frac{1}{2}\mu_{O_2}[gas] \quad (5)$$

The last inequality gives a rough upper limit of μ_O to be -4.85 eV. The first three inequalities, combined with the following stoichiometric equation:

$$E_{[SIO]1[SMO]1} = 4\mu_{Sr}[superlattice] + 2\mu_{Ir}[superlattice] + 2\mu_{Mn}[superlattice] + 12\mu_{O}[superlattice]$$

produce a lower bound of μ_O as -7.88 eV. Consequently, we can estimate the lower and upper bounds of the oxygen vacancy formation energy at the three different sites in Figure 6(b), which are listed in Supplementary Table 1 below.

It is noted that the relative energy difference of the same type of defect species at distinct positions does not rely on the exact value of μ. As shown in Supplementary Table 1, the oxygen vacancy in the MnO_2 layer is energetically more favorable by 0.46 eV than that in the SrO layer, and by 0.57 eV than that in the IrO_2. These results suggest that the MnO_2 plane is energetically more favorable to host the oxygen vacancy after ion transfer.

Supplementary Table 1: Calculated oxygen vacancy formation energy at different sites

Oxygen Vacancy DFE	O1 (SrO Layer)	O2 (IrO_2 Layer)	O3 (MnO_2 Layer)
Lower Limit (eV)	-0.46	-0.35	-0.92
Upper Limit (eV)	2.57	2.68	2.11

Regarding the hydrogen interstitial, we followed the same procedure and used the chemical potential of hydrogen gas to obtain an upper bound of μ_H as -3.26 eV. By using it in Supplementary Equation (1), the lower bound of the interstitial hydrogen E^f was calculated, which ranges from 2.74 eV to 2.96 eV depending on the final configuration (local minimum) into which the interstitial was optimized. In all 18 cases in Supplementary Figure 13(a), we found that the hydrogen atom favors pairing with an oxygen, forming an interstitial dumbbell. The specific interstitial configuration can be categorized by the dumbbell orientation combined with the attached oxygen. We found that the [100]/[010] dumbbell attached to O2 ([100]-O2) and [001] attached to O3 ([001]-O3) have the lowest relative DFEs of the interstitial hydrogen. The DFEs of [100]-O1 and [100]-O3 dumbbell are slightly higher by 0.01 eV to 0.03 eV. The [001]-O2 has the highest DFE and is roughly 0.2 eV larger than that of the [100]-O2 dumbbell. All the orientations mentioned above are approximate, within 30 degrees of the Miller index directions, as illustrated in Supplementary Figure 13(b). These results show that the formation energy of hydrogen interstitial also depends on the local chemical environment. Moreover, the difference of formation

energies of hydrogen interstitials at different sites seems to be smaller than that of oxygen vacancies at different sites.

It is noted that the concentration of the ionic species that was considered in these DFT calculations (one oxygen vacancy or one hydrogen interstitial in the supercell of $Sr_4Ir_2Mn_2O_{12}$) is likely to be much lower than the real concentration in phase B of the superlattices, which can be roughly deduced from experimental results (see Discussion section of main text). Nevertheless, the DFT calculations suggest that the formation energies of ionic species are different and depend on the local chemical environment. Therefore, given the superlattice structure with unit-cell layering, ordering of oxygen vacancies or hydrogen interstitials is likely to develop in phase B upon massive ion transfer. This transition resembles the topotactic phase transition in perovskite oxides (such as $H_xSrCoO_{3-\delta}$)[1] although neither $La_{1-x}Sr_xMnO_3$ nor $SrIrO_3$ show this topotactic phase transition at room-temperature under ILG.

There are several possible mechanisms to account for the difference in formation energy due to the atomically layered structure. First, it may be correlated to the different strength of the metal-oxygen bonds (e.g., Mn-O-Mn, Ir-O-Mn and Ir-O-Ir) for different oxygen anions (and therefore O-H dumbbell). Second, it may also be correlated to the different deformation/rotation of oxygen octahedra[22]. In fact, our DFT calculations have already shown the different octahedral rotations in iridate and manganate layers (Figure 6(a)). Further experimental studies on additional material systems, along with theoretical studies, are necessary for a more comprehensive understanding of the factors governing these structural transformations.

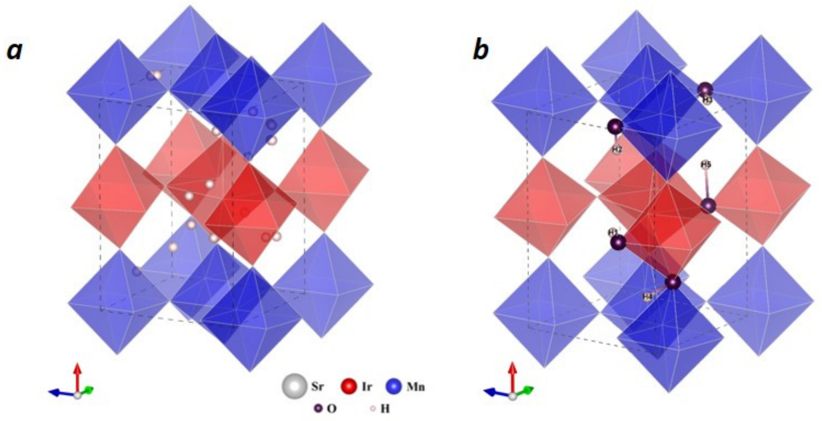

Supplementary Figure 13 | Illustrations of H interstitials in the superlattice considered in DFT calculations. (a) Initial 18 hydrogen interstitials sites, generated via Voronoi analysis. (b) Five types of O-H dumbbells obtained after structure relaxation. H1~H5 corresponds to [100]-O2, [001]-O3, [100]-O3, [100]-O1, [001]-O2, respectively.

Supplementary Note 14: Certain trade names and company products are identified to specify adequately the experimental procedure. In no case does such identification imply recommendation or endorsement by the National Institute of Standards and Technology, nor does it imply that the products are necessarily the best for the purpose.

Supplementary References

1 Lu, N. *et al.* Electric-field control of tri-state phase transformation with a selective dual-ion switch. *Nature* **546**, 124, (2017).

2 De Souza, R. A. & Kilner, J. A. Oxygen transport in $La_{1-x}Sr_xMn_{1-y}Co_yO_{3\pm\delta}$ perovskites: Part I. Oxygen tracer diffusion. *Solid State Ionics* **106**, 175-187, (1998).

3 Grutter, A. J. *et al.* Reversible control of magnetism in $La_{0.67}Sr_{0.33}MnO_3$ through chemically-induced oxygen migration. *Applied Physics Letters* **108**, 082405, (2016).

4 Gilbert, D. A. *et al.* Ionic tuning of cobaltites at the nanoscale. *Physical Review Materials* **2**, 104402, (2018).

5 Clancy, J. P. *et al.* Spin-orbit coupling in iridium-based 5*d* compounds probed by x-ray absorption spectroscopy. *Physical Review B* **86**, 195131, (2012).

6 Laguna-Marco, M. A. *et al.* Electronic structure, local magnetism, and spin-orbit effects of Ir(IV)-, Ir(V)-, and Ir(VI)-based compounds. *Physical Review B* **91**, 214433, (2015).

7 Yi, D. *et al.* Tuning Perpendicular Magnetic Anisotropy by Oxygen Octahedral Rotations in $La_{1-x}Sr_xMnO_3/SrIrO_3$ Superlattices. *Physical Review Letters* **119**, 077201, (2017).

8 Hohenberg, P. & Kohn, W. Inhomogeneous electron gas. *Physical review* **136**, B864, (1964).

9 Kohn, W. & Sham, L. J. Self-consistent equations including exchange and correlation effects. *Physical review* **140**, A1133, (1965).

10 Kresse, G. & Hafner, J. Ab initio molecular dynamics for liquid metals. *Physical Review B* **47**, 558, (1993).

11 Kresse, G. & Furthmüller, J. Efficiency of ab-initio total energy calculations for metals and semiconductors using a plane-wave basis set. *Computational materials science* **6**, 15-50, (1996).

12 Perdew, J. P. *et al.* Restoring the density-gradient expansion for exchange in solids and surfaces. *Physical Review Letters* **100**, (2008).

13 Blöchl, P. E. Projector augmented-wave method. *Physical review B* **50**, 17953, (1994).

14 Dudarev, S. L., Botton, G. A., Savrasov, S. Y., Humphreys, C. J. & Sutton, A. P. Electron-energy-loss spectra and the structural stability of nickel oxide: An LSDA+U study. *Physical Review B* **57**, 1505-1509, (1998).

15 Picozzi, S. *et al.* Oxygen vacancies and induced changes in the electronic and magnetic structures of $La_{0.66}Sr_{0.33}MnO_3$: A combined ab initio and photoemission study. *Physical Review B* **75**, 094418, (2007).

16 Arita, R., Kunes, J., Kozhevnikov, A. V., Eguiluz, A. G. & Imada, M. Ab initio Studies on the Interplay between Spin-Orbit Interaction and Coulomb Correlation in Sr_2IrO_4 and Ba_2IrO_4. *Physical Review Letters* **108**, (2012).

17 Zhang, L. P. *et al.* Oxygen vacancy diffusion in bulk $SrTiO_3$ from density functional theory calculations. *Computational Materials Science* **118**, 309-315, (2016).

18 Dasa, T. R., Hao, L., Yang, J., Liu, J. & Xu, H. Strain effects on structural and magnetic properties of $SrIrO_3/SrTiO_3$ superlattice. *Materials Today Physics* **4**, 43-49, (2018).

19 Okamoto, S. *et al.* Charge Transfer in Iridate-Manganite Superlattices. *Nano Lett* **17**, 2126-2130, (2017).

20 Ong, S. P. *et al.* Python Materials Genomics (pymatgen): A robust, open-source python library for materials analysis. *Computational Materials Science* **68**, 314-319, (2013).

21 Freysoldt, C. *et al.* First-principles calculations for point defects in solids. *Rev Mod Phys* **86**, (2014).

22 Aschauer, U., Pfenninger, R., Selbach, S. M., Grande, T. & Spaldin, N. A. Strain-controlled oxygen vacancy formation and ordering in $CaMnO_3$. *Physical Review B* **88**, 054111, (2013).
